# Nanoparticles in influenza research: a bibliometric analysis of global trends (2005 - 2025)

**DOI:** 10.3389/fimmu.2026.1715271

**Published:** 2026-01-21

**Authors:** Yezi Zhang, Xi Lin, Lingling Wen, Jiaona Zhang, Jian Lin, Xinghua Liu, Yuxiang Liu, Siyuan Xu, Xingdong Lin, Zhizhun Mo

**Affiliations:** 1 The Third Clinical Medical College of Guangzhou University of Chinese Medicine, Guangzhou, China; 2 Shenzhen Traditional Chinese Medicine Hospital, The Fourth Clinical Medical College of Guangzhou University of Chinese Medicine, Shenzhen, China; 3 Guangzhou University of Chinese Medicine Science and Technology Innovation Center, Guangzhou, China; 4 The Third Affiliated Hospital of Guangzhou University of Chinese Medicine, Guangzhou, China

**Keywords:** bibliometric, CiteSpace, influenza, nanoparticles, VOSviewer

## Abstract

**Background:**

Influenza viruses cause approximately 3 to 5 million severe cases and tens of thousands of deaths globally each year, posing a continuous threat to public health. Nanoparticles with superior biocompatibility, combined with traditional Chinese herbal medicine could offer promising avenues to enhance therapeutic efficacy, improve targeting precision, and address multidrug resistance. This study aims to provide a comprehensive review of the current status and future directions of nanoparticle applications in influenza research, thereby providing more effective and convenient solutions for influenza prevention and control.

**Methods:**

A publication search was performed using Web of Science Core Collection, Scopus, and PubMed from 2005 to 2025 to improve the completeness of publications collection. Bibliometric analyses performed using Bibliometrix, CiteSpace, and VOSviewer software to compensate for the differences in their respective algorithms. The analysis by evaluating publication quantity, Citation Counts, and Co-authorship status to find the core institutions, journals, and key authors. Explore the research hotspots of nanoparticles and influenza through keyword analysis.

**Result:**

A total of 3,478 relevant publications were identified. The United States and China were the primary contributors in terms of publication volume, with the Chinese Academy of Sciences leading in institutional contributions. Vaccines emerged as the top-performing journal in this field. Masaru Kanekiyo’s article had the highest local citation count, and he was identified as the author with the greatest individual contribution. Keyword analysis revealed that optimizing delivery systems and advancing vaccine development are central research priorities. Notably, bibliometric analysis revealed that besides metal-based nanoparticles, viral-like particles, polymer nanoparticles, and lipid nanoparticles in the field of delivery system research, herbal small-molecule nanoparticle delivery system has garnered significant attention due to the promise for future clinical applications.

**Conclusion:**

Research on nanoparticles in influenza is rapidly advancing, with a primary focus on their application in vaccine development and delivery optimization. The field is also exploring nanoparticles from various sources. Future research should bridge basic mechanistic studies with clinical applications to enhance their impact.

## 1 Introduction

Among the four influenza viruses—A, B, C, and D—identified in nature, the influenza A virus (IAV) has the most profound impact on humans due to its high morbidity and mortality rates associated with seasonal epidemics. At least five pandemics have been documented since the 20th century: the 1918 “Spanish flu”, the 1957 “Asian flu”, the 1968 “Hong Kong flu,” the 1977 “Russian flu”, and the 2009 “Swine flu” (1–3). Subsequently, H1N1 and H3N2 influenza strains have continued to circulate as seasonal influenza, posing significant threats to public health (2). Each year, approximately one billion people contract seasonal influenza, with 3 to 5 million severe cases (4).

Nanoparticles (NPs) < 1000 nm exhibit distinct physicochemical properties compared to their bulk counterparts, making them valuable in medical applications. The increasing demand for new drug development, together with the nanoscale nature of cellular components, has promoted the use of NPs (5). NPs are highly sensitive analytical tools for *in vivo* and *in vitro* diagnosis. They can enrich and amplify biomarkers and inhibit their degradation (6). Their small size, large surface area, ability to load multiple therapeutic biomolecules, and tunable surface chemical properties have allow NPs to penetrate biological barriers, enabling precise delivery (7). These features have catalyzed a surge in research on NPs in influenza vaccine development, targeted therapy, and detection (8, 9). In addition, due to the properties of nanotechnology, preparation of herbal small-molecule nanoparticles has attracted significant attention to overcome the shortcomings of low bioavailability, insufficient targeting ability, restricted administration routes, and poor water solubility of herbs and their natural compounds (10).

Bibliometrics is an effective tool for analyzing publication distribution and trends within databases (11). This study aims to perform a bibliometric evaluation of the relationship between the NPs applications and influenza. It is intended to expand researchers’ knowledge, find the rapid assessment of emerging research hotspots, and visualize future trends (12).

## 2 Materials and methods

### 2.1 Data source and search method

Data sources for this study include the Web of Science Core Collection (WoSCC), PubMed, and Scopus. To minimize temporal bias, data collection was completed on September 11, 2025. A combination strategy utilizing Medical Subject Headings (MeSH) terms and keywords was employed, specifically including terms such as exosome*, nanovesicle*, nanoparticle*, “extracellular vesicle*”, influenza, and other relevant keywords. The final search expression in the PubMed database was: ((exosome*[Title] OR nanovesicle*[Title] OR nanoparticle*[Title] OR “extracellular vesicle*”[Title] OR EVs[Title]) OR (exosome*[Abstract] OR nanovesicle*[Abstract] OR nanoparticle*[Abstract] OR “extracellular vesicle*”[Abstract] OR EVs[Abstract]) OR (exosome*[MeSH Terms] OR nanovesicle*[MeSH Terms] OR nanoparticle*[MeSH Terms] OR “extracellular vesicle*”[MeSH Terms] OR EVs[MeSH Terms])) AND (influenza [Title] OR influenza[Abstract] OR influenza[MeSH Terms]). After refining the search terms, publication screening was conducted in the WoSCC and Scopus databases for studies published between 2005 and 2025. The search was restricted to English-language research articles and review papers. A total of 1,327, 2,316, and 2,420 articles were retrieved from PubMed, WoSCC, and Scopus, respectively. Data were exported from the collected databases, including full records and bibliographic details (titles, keywords, authors, institutions, references, etc.).

**Figure 1:**
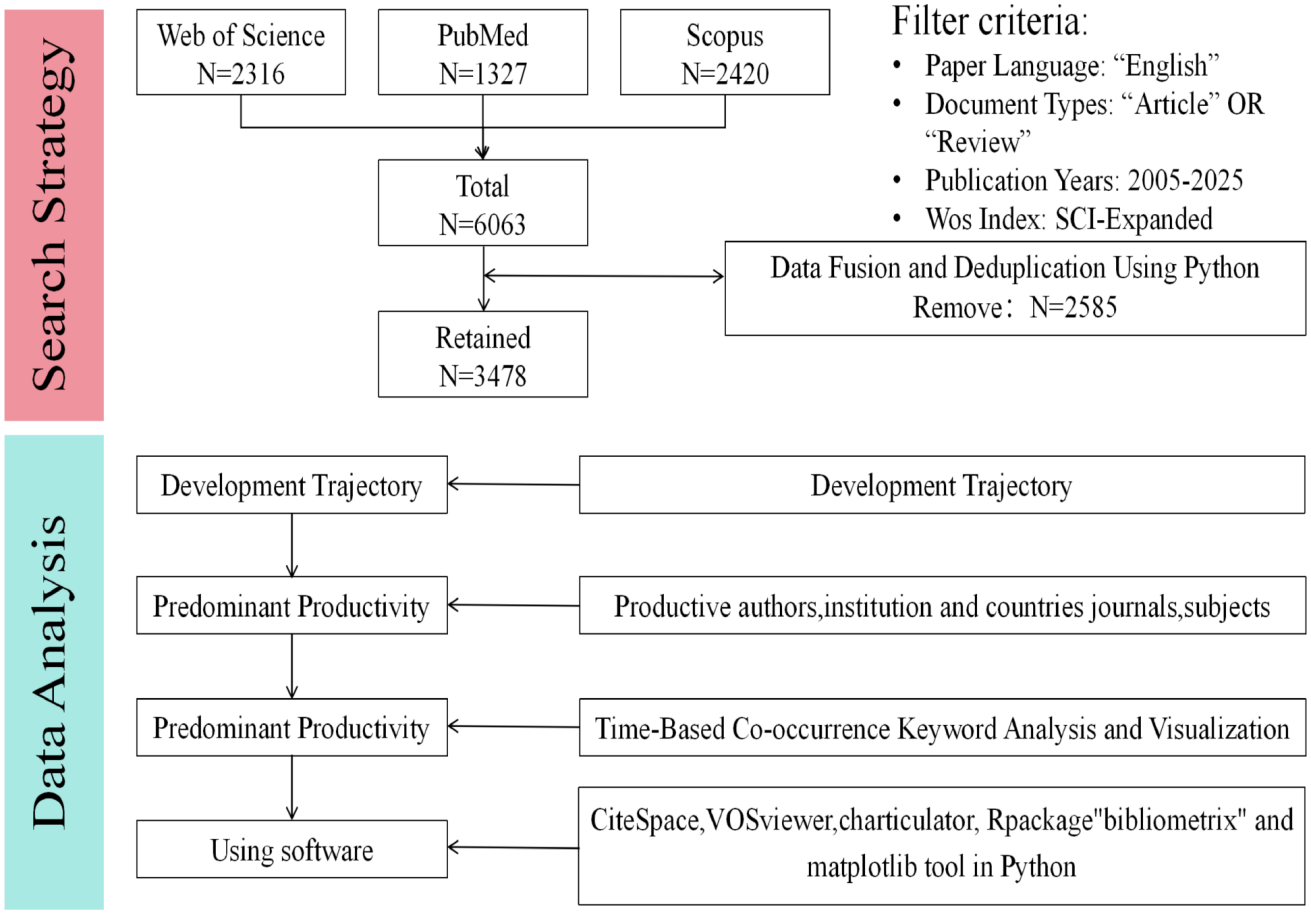
Search strategy and research framework.

To achieve the unification of data, this study utilized Python (version 3.11) to convert Scopus CSV files into plain text format consistent with WoSCC and PubMed. Data cleaning was also performed using Python (version 3.11). The key steps included: deduplication based on DOI; removal of records where the author field contained “[Anonymous]”; exclusion of virtual institutions such as “Egyptian Knowledge Bank (EKB)”; and the merging and standardization of duplicate institution names. A total of 3,478 articles were ultimately included for subsequent bibliometric analysis (**Figure 1**). CiteSpace, VOSviewer and Bibliometrix were then used to analyze annual outputs, countries, institutions, authors, co-cited references, and keywords.

### 2.2 Data analysis

Bibliometrix, a bibliometric analysis tool developed using R software, was employed to conduct analyses on the H-index of authors and local citation of references. CiteSpace and VOSviewer were used for visualizing the bibliometric data and highlighting the strength of connections between raw data points. Additionally, Python’s matplotlib tool were employed for plotting.

## 3 Result

### 3.1 Analysis of annual publication quantity and growth trend

**Figure 2:**
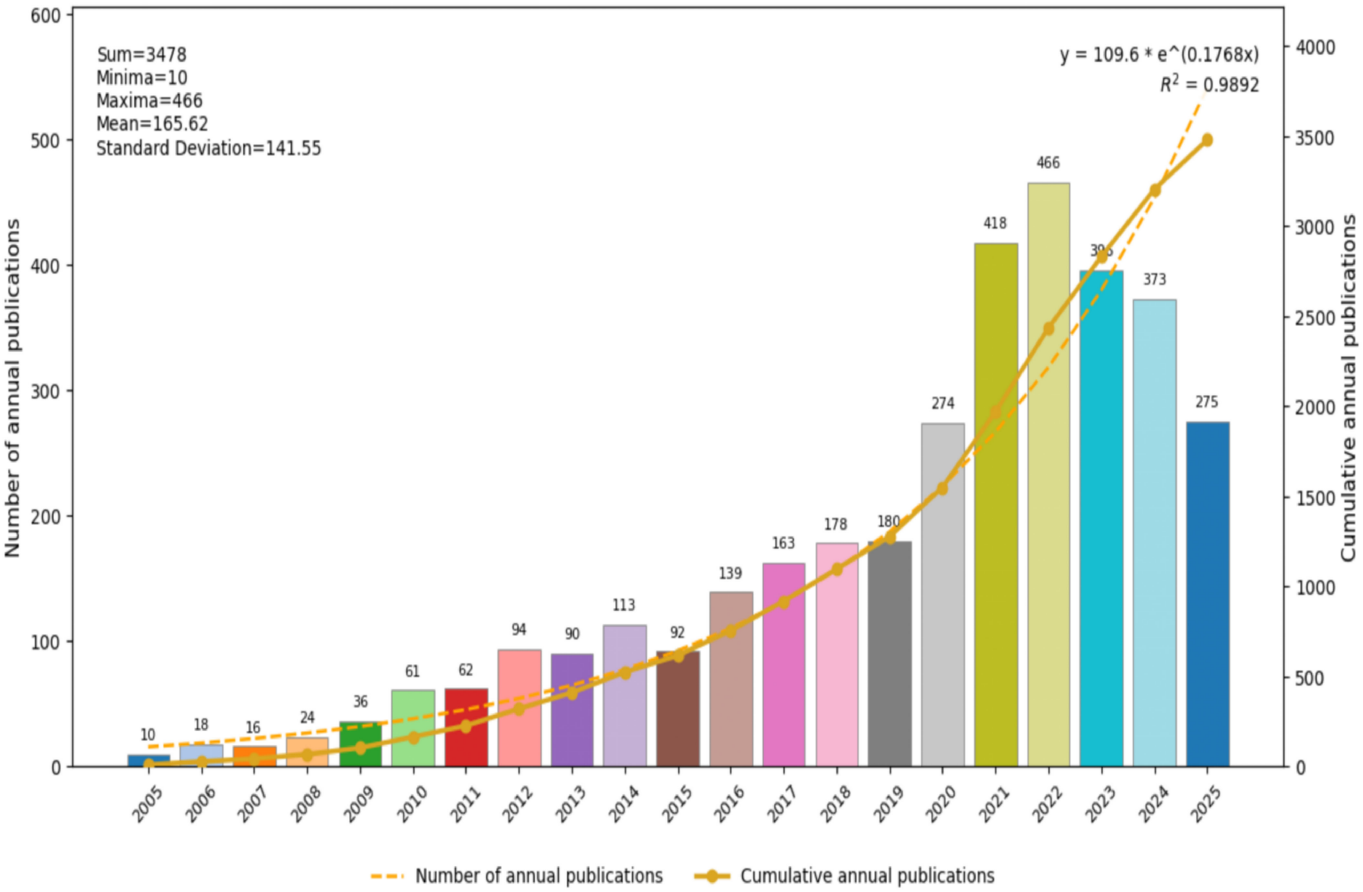
Annual distribution, cumulative growth, and statistical fitting characteristics of publication volume from 2005 to 2025 on nanoparticles and influenza research.

Publications related to this topic have shown a clear three-phase growth pattern, with growth observed from 2005 to September 11, 2025. The period from 2005 to 2009 represented an early exploration and development phase of the technology, with an average of only 13 papers published annually. Key growth points corresponded closely with global public health events. Following the 2009 H1N1 influenza pandemic, a significant increase in annual publications from 2010 to 2019 marked the entry of research into an accelerated development phase, signaling the transition to more systematic research. While the 2020 COVID-19 pandemic triggered an explosive rise in nucleic acid NPs vaccine research in the period from 2019 to 2025, with publications jumping from 274 in 2020 to 418 in 2021. Publication volume had remained high since, highlighting the powerful catalytic effect of major infectious disease crises on technological innovation (**Figure 2**).

### 3.2 Analysis of countries/regions and institutions

**Figure 3:**
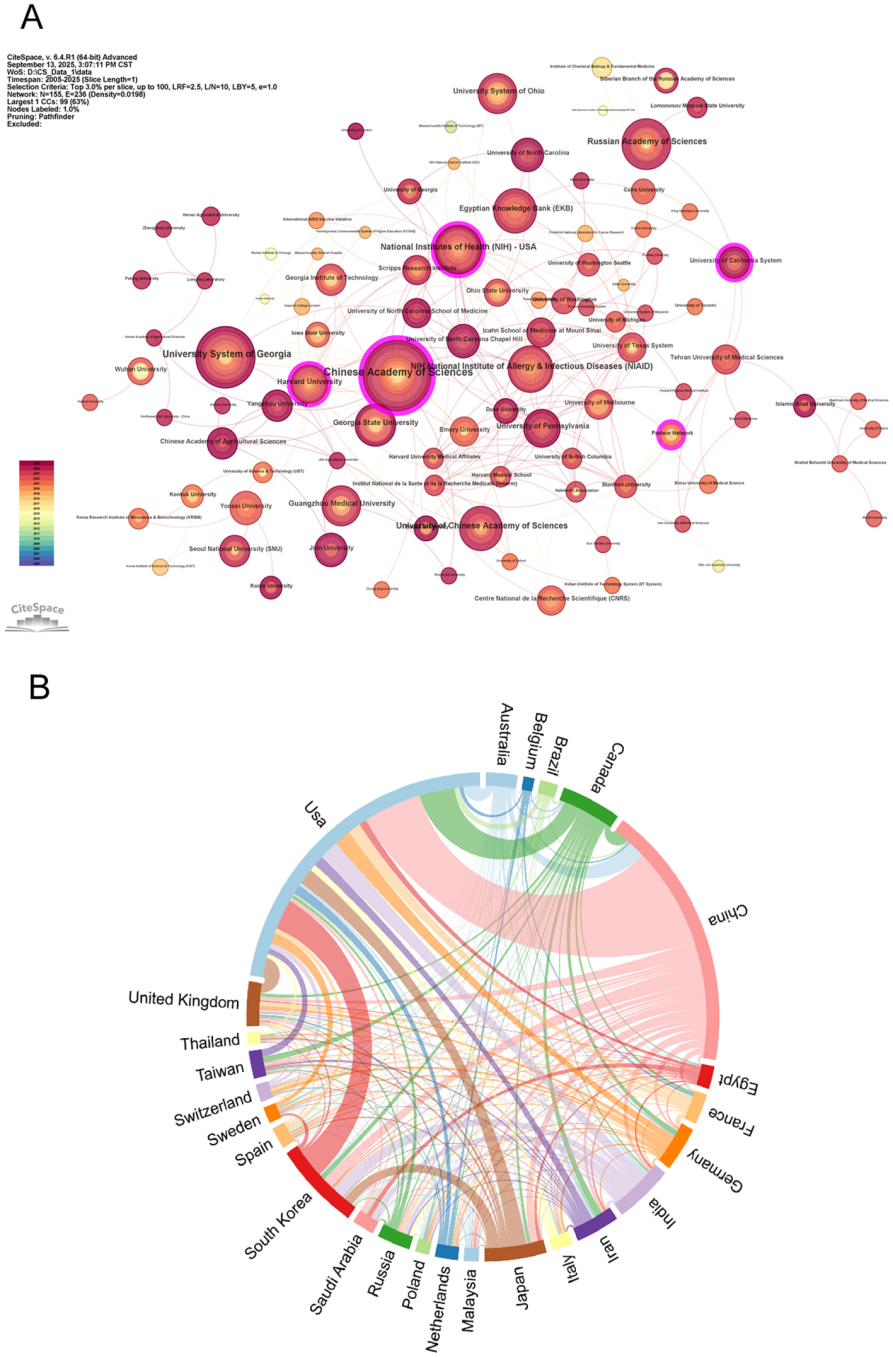
Contribution and collaboration among countries/regions and institutions on nanoparticles and influenza research. **(A)** The cooperation network of institutions. **(B)** Visual network analysis of country relations. Node size indicates citation frequency, line width shows interaction strength, and colors represent clusters.

An analysis of international collaboration and publication trends within the academic field revealed that, the United States led both in the number of 962 articles and 35,485 citations. China followed closely with 771 publications. It indicated the strong productivity and active involvement from American and Chinese scholars (**Table 1**). The collaborative network in this field was multi-centered and multi-layered. The Centrality of Chinese Academy of Sciences was 0.3 and National Institutes of Health occupying was 0.12, which indicated that they occupied central positions. This dominance likely resulted from their extensive research resources and broad collaborative networks, which enable the construction of large-scale projects (**Figure 3A**). The total link strength of the United States was 396 and China was 249, occupied central positions in global cooperation. Overall, the United States and China served as core nodes directing global cooperation. The other Countries with total link strength between 100 and 200 were engaged in various collaborative projects based on their research capabilities and specific needs, collectively advancing the development and progress of the academic domain (**Figure 3B**).

**Table 1:** The top 11 most production institutions and countries/regions on nanoparticles and influenza research.

Rank	Institution	Country/ region	Article counts	Centrality	Rank	Country/ region	Article counts	Citations
1	Chinese Academy of Sciences	China	93	0.3	1	USA	962	35485
2	University System of Georgia	USA	77	0.08	2	China	771	18515
3	National Institutes of Health	USA	47	0.12	3	South Korea	249	6456
4	Russian Academy of Sciences	Russian	46	0.08	4	India	236	4015
5	University of Chinese Academy of Sciences	China	38	0.02	5	Canada	172	8013
6	NIH National Institute of Allergy & Infectious Diseases	USA	37	0.03	6	Japan	167	5078
7	Georgia State University	USA	33	0	7	Iran	149	2868
8	Egyptian Knowledge Bank	Egypt	33	0.05	8	Germany	127	5570
9	Harvard University	USA	31	0.15	9	Australia	126	2866
10	Guangzhou Medical University	China	30	0	10	UK	95	3633
11	University of Pennsylvania	USA	30	0.06	11	France	95	2556

### 3.3 Analysis of authors and co-cited authors

**Figure 4:**
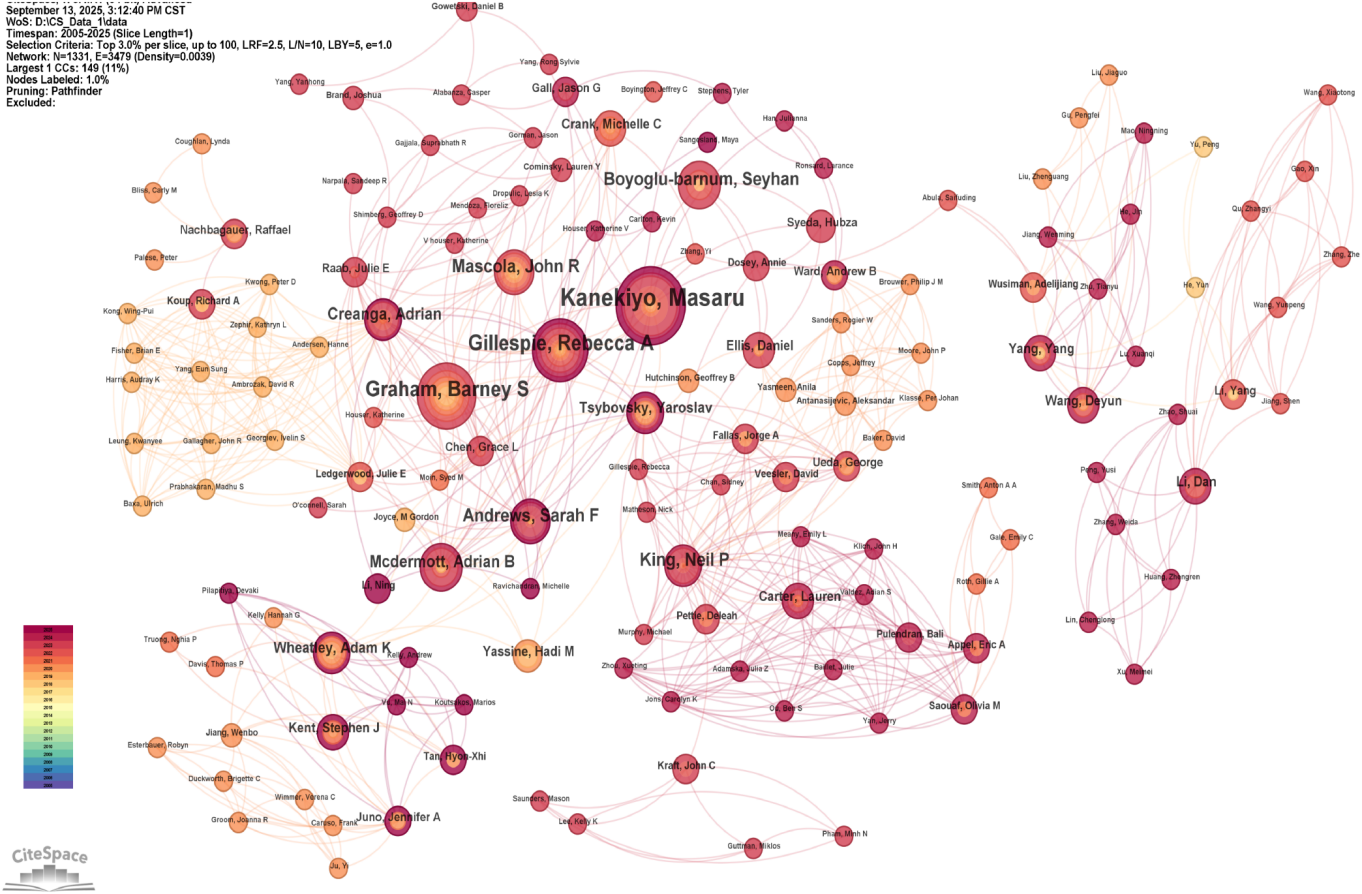
Visual network analysis of author contributions and collaborations on nanoparticles and influenza research. Node size reflects the number of articles, line width indicates interaction strength, and colors represent different clusters.

Based on the analysis of authors and co-cited authors in the field of NPs and influenza research, Masaru Kanekiyo ranked first with 36 papers and 2,263 citations and had 238 high co-citation counts, demonstrating his substantial academic influence and widespread recognition in this field (**Table 2**). Norbert Pardi had 2234 citations, 20 papers and the highest 336 co-citation counts. Yinghua Li had 1013 citations, 25 papers and second highest 304 co-citation counts. The finding indicated that these three authors with other authors in the field of NPs and influenza research had a degree of association and exert significant group influence (**Table 2**). Notably, Masaru Kanekiyo with 36 papers, Barney S Graham with 22 papers, and Rebecca A Gillespie with 21 papers, have high productivity and frequently collaborate with other scholars, which forms the core of the research network. This suggested that regular collaboration among authors not only advances their individual academic careers, driving critical advances in the field of NPs and influenza (**Figure 4**).

**Table 2:** The top 11 most productive authors and co-cited authors on nanoparticles and influenza research.

Rank	Author	Article counts	Citations	H-index	Rank	Co-cited author	Co-citation
1	Masaru Kanekiyo	36	2263	37	1	Norbert Pardi	336
2	Baozhong Wang	33	814	29	2	Yinghua Li	304
3	Bing Zhu	28	1271	29	3	Florian Krammer	284
4	Yinghua Li	25	1013	24	4	Masaru Kanekiyo	238
5	Barney S Graham	22	1845	119	5	Ye Wang	217
6	Rebecca A Gillespie	21	344	21	6	Ji Wang	198
7	Norbert Pardi	20	2234	56	7	Yun Zhang	195
8	Wandi Zhu	18	302	16	8	Lei Deng	188
9	Chunhong Dong	18	270	19	9	Jaeyoung Kim	180
10	Mingqi Zhao	17	864	31	10	Santosh Dhakal	164
11	Tiantian Xu	17	854	25	11	Ying Li	164

### 3.4 Journal analysis

**Figure 5:**
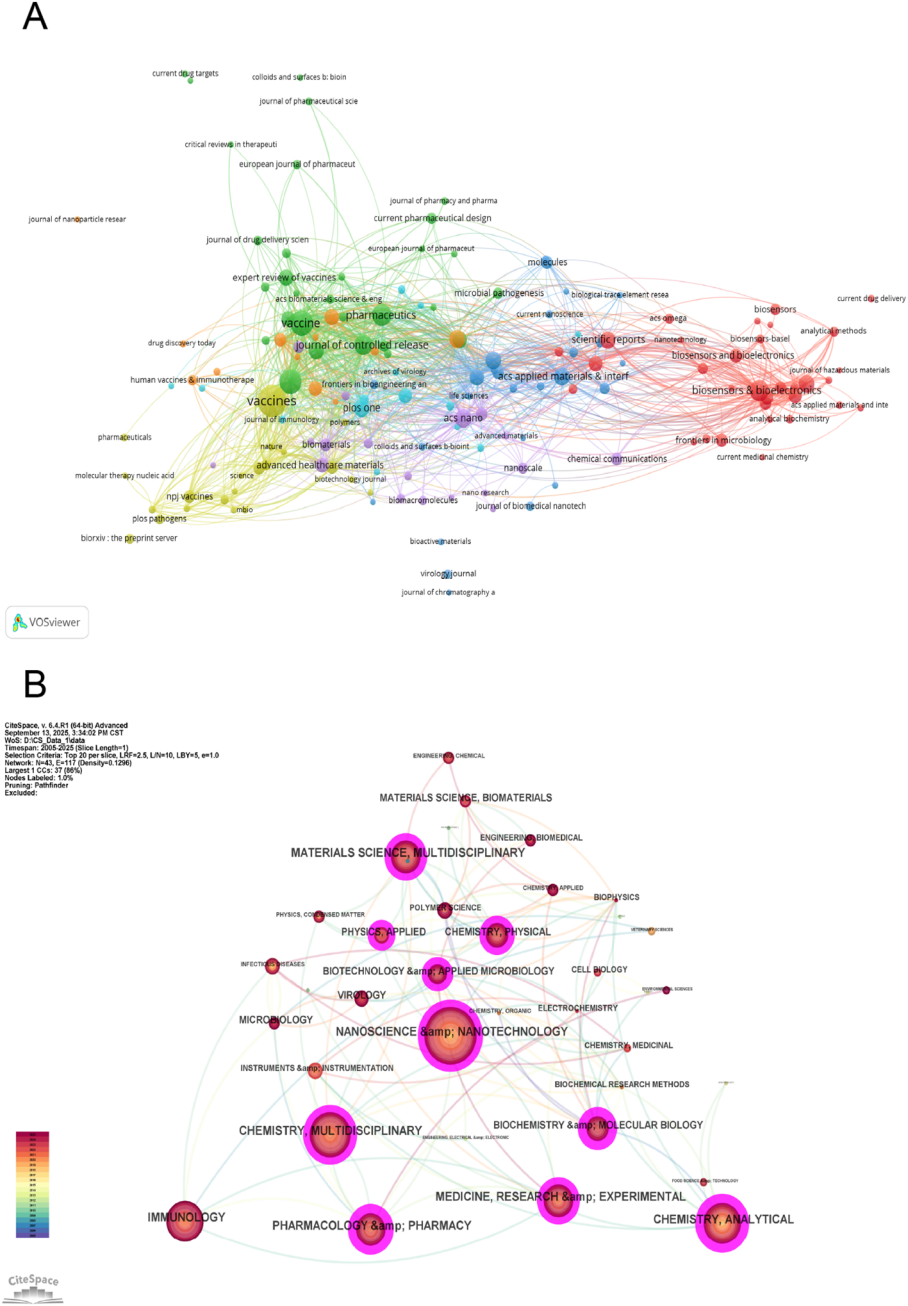
The most productive and influential **(A)** journals and **(B)** disciplines in research on nanoparticle applications for influenza. Node size reflects the number of articles, and colors represent different clusters.

Several journals with more publications among the top 10 were classified in the JCR Q1 quartile. MDPI’s Vaccines led with 119 publications. Elsevier’s Vaccine and Frontiers in Immunology followed in second and third place, with 79 and 73 publications, respectively, demonstrating their active involvement in related research areas. Several highly cited journals among the top 10 boasted high impact factors. High impact factors reflected the activity level and rapid development of the field of NPs and influenza (**Table 3**). The disciplinary co-occurrence network diagram indicated that Nanoscience & Nanotechnology with 0.18 Centrality is the most important bridge discipline in this field of NPs and influenza research, exhibiting strong co-occurrence with Immunology, Pharmacology & Pharmacy, and Materials Science. This reflected deep interdisciplinary connections across nanotechnology, chemistry, biology, medicine, and engineering (**Figure 5**).

**Table 3:** Top 10 journals and co-cited journals publishing research on nanoparticles and influenza.

Rank	Source	Article counts	IF	JCR	Rank	Co-cited journal	Citation	IF	JCR
1	Vaccines (MDPI)	119	3.4	Q2	1	Nature	1242	48.5	Q1
2	Vaccine (Elsevier)	79	3.5	Q2	2	Proceedings of the National Academy of Sciences	1226	9.4	Q1
3	Frontiers in Immunology	73	5.9	Q1	3	PLOS One	1223	2.6	Q2
4	Pharmaceutics	57	5.5	Q1	4	Science	1133	45.8	Q1
5	Biosensors & Bioelectronics	54	10.5	Q1	5	Vaccine (Elsevier)	1132	3.5	Q2
6	Journal of Controlled Release	50	11.5	QI	6	Scientific Reports	822	3.9	Q1
7	ACS Nano	40	16	Q1	7	Journal of Virology	461	3.8	Q2
8	International Journal of Nanomedicine	39	6.5	Q1	8	ACS Nano	452	16	Q1
9	ACS applied Materials & interfaces	39	8.5	Q1	9	Nature Communications	236	15.7	Q1
10	PLOS One	38	2.6	Q2	10	Frontiers in Immunology	232	5.9	Q1

### 3.5 Local citation analysis

**Figure 6:**
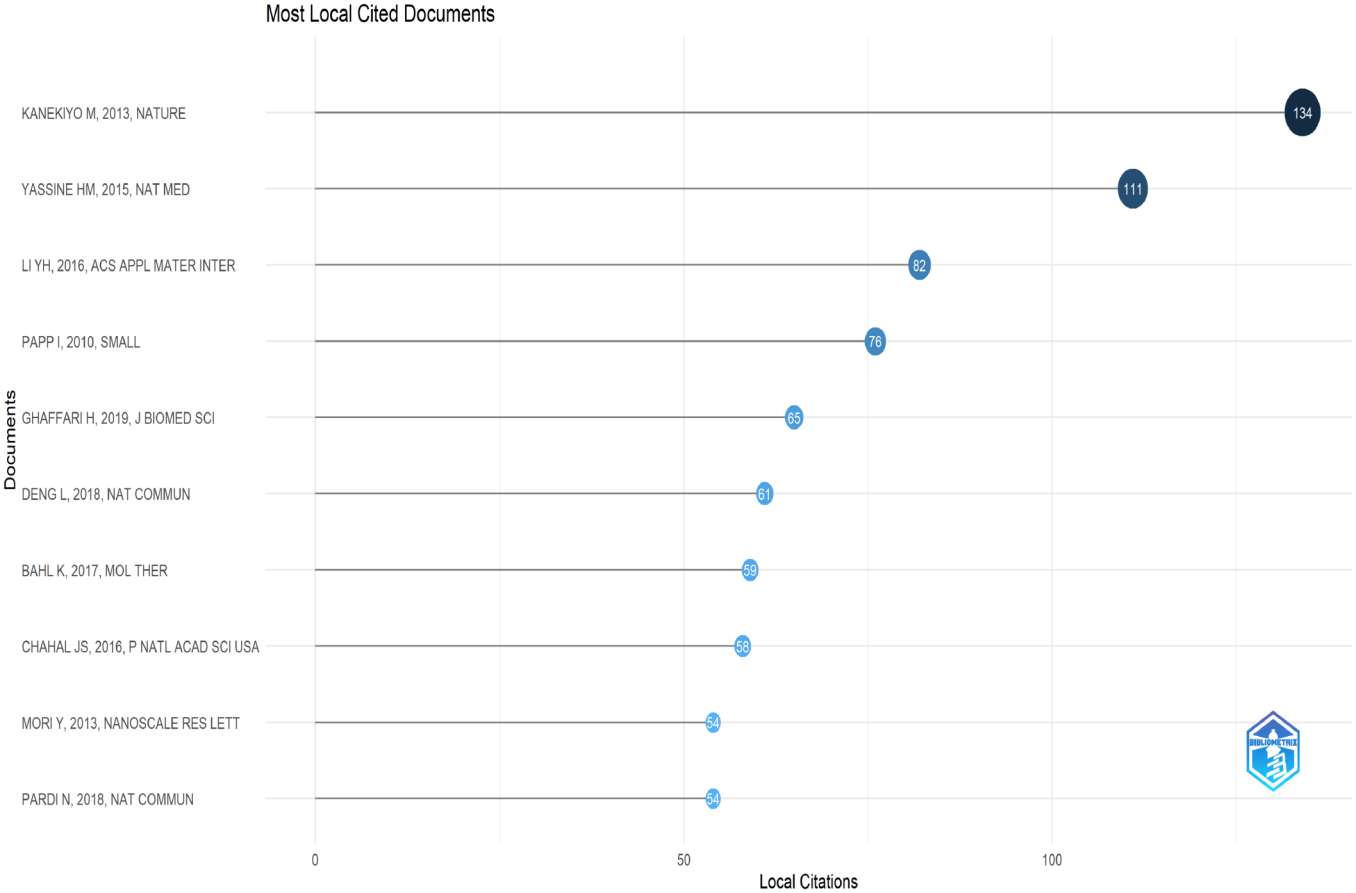
Top 10 most cited local articles on nanoparticles and influenza research. Node size and line length reflect the number of references.

This research highlighted the 10 most cited local papers. NPs vaccine designs were generally divided into two categories. The first category of NPs vaccines based on protein structure design. Masaru Kanekiyo’s pioneering work, published in Nature, employed the hemagglutinin (HA) protein structure to design HA NPs, significantly enhancing immune efficacy and breadth against influenza viruses (13). It had the highest Local Citation Score (LCS) of 134. Barney S. Graham’s structure-based design of an HA stem-specific antigen provided cross-subtype protection in ferrets and mice (14). Lei Deng combined the stable HA stem domain with the M2e tetramer from IAV protein in a bilayer protein NPs, which enhanced the efficacy and breadth of influenza vaccine protection (15). The second category of vaccines based on mRNA-LNPs technology. Kapil Bahl and Norbert Pardi explored nucleoside-modified mRNA-LNPs platforms for influenza vaccines (16, 17). These highly cited studies showcased strategies for developing broad-spectrum influenza vaccines and antiviral nanomedicines utilizing nanotechnology (**Figure 6**).

These studies on the synergistic delivery of NPs and drugs provided novel approaches for developing new antiviral drugs. Ilona Papp functionalized gold NPs (AuNPs) using an efficient method to synthesize sialic acid-terminated glycerol dendrimers, demonstrating potent activity in inhibiting influenza virus infection (18). Yasutaka Mori’s silver NPs (AgNPs)/chitosan composite exhibited significant anti-H1N1 IAV activity (19). Building on this, Yinghua Li further explored a synergistic delivery system combining AgNPs with oseltamivir (20). This progress offered critical technical support in combating drug-resistant viral strains and emerging epidemic variants (**Figure 6**).

### 3.6 Reference analysis

**Figure 7:**
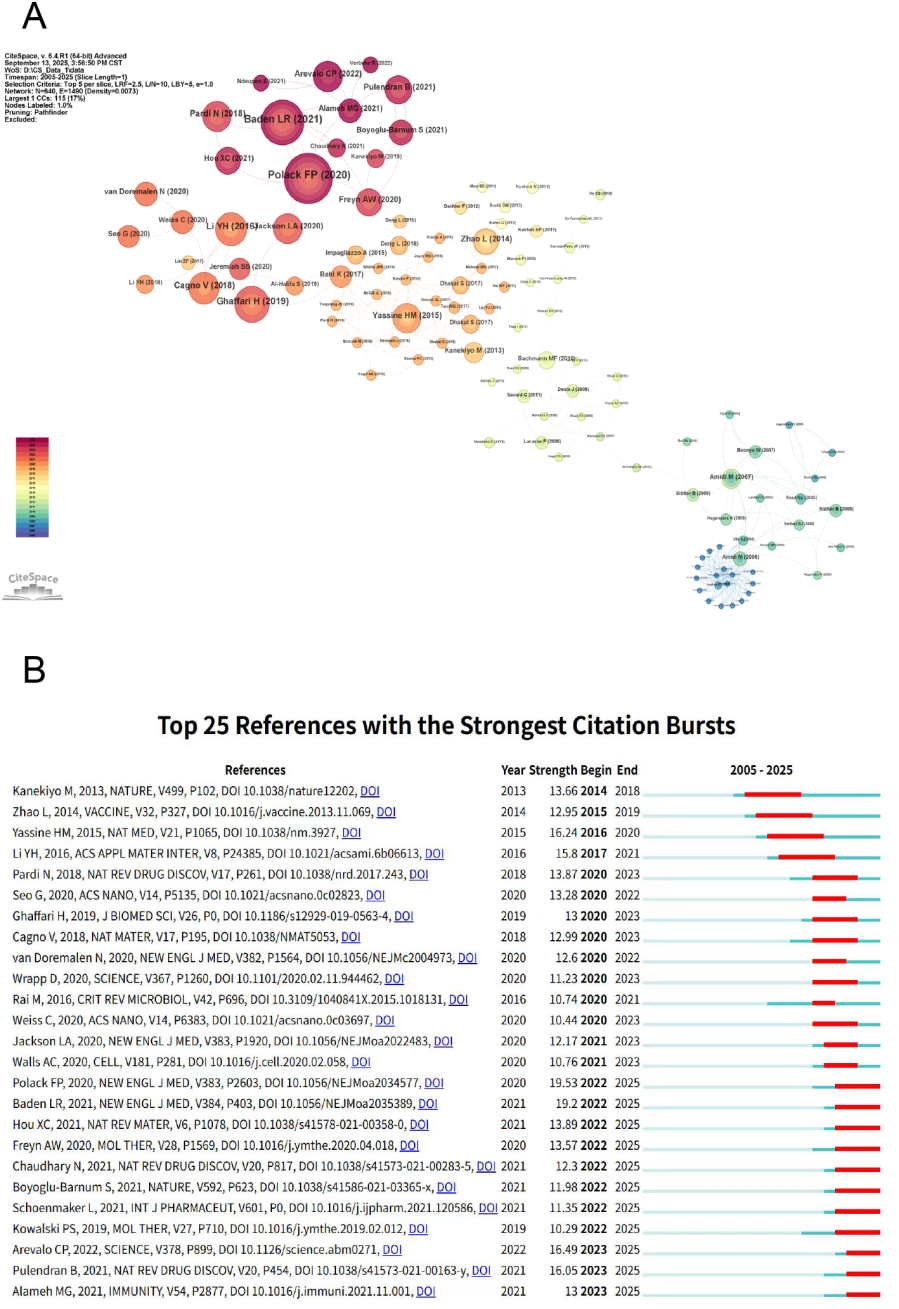
Visual analysis of references on nanoparticles and influenza research. **(A)** Network map of co-cited references. The citation frequency of references are represented as nodes, with lines connecting nodes to indicate co-citation relationships, and different colors distinguish clusters. **(B)** Top 25 references with the strongest citation bursts. The red line segment representing the duration of high-frequency occurrences and the “strength” indicating the frequency of references.

Citation analysis of publications was an effective method for identifying high-impact articles within specific fields. Cluster 1 (red) focused on the development of influenza virus vaccines, with an emphasis on demonstrating their safety and providing long-term immune protection in clinical trials. Cluster 2 (orange) addressed optimizing immune response mechanisms and innovated delivery technologies to combat immune escape in highly mutated viruses and meet clinical treatment needs. Cluster 3 (yellow) concentrated on the preparation and development of various NPs vaccines. Cluster 4 (blue-green) explored the optimal strategies for administering different vaccine types using distinct inoculation methods (**Figure 7A**).

This study also identified the top 25 references exhibiting the strongest citation surge effects, where “Begin” indicates the start of the burst and “Strength” reflects its intensity. Notably, the longest-running surge stems from was Masaru Kanekiyo’s team, published in Nature publication introduced self-assembling NPs leveraging protein structural knowledge to induce broader and more potent immune responses than traditional influenza vaccines (13). The study of Fernando P. Polack showed the strongest surge intensity of 19.53, evaluated the safety and clinical protective efficacy of a nucleoside-modified RNA vaccine encapsulated in LNPs, laying the foundation for subsequent experiments (21). This suggested that protein self-assembling NPs and nucleoside-modified RNA vaccine encapsulated in LNPs had a significant and lasting impact in the field of NPs and influenza (**Figure 7B**).

### 3.7 Keyword analysis

**Figure 8:**
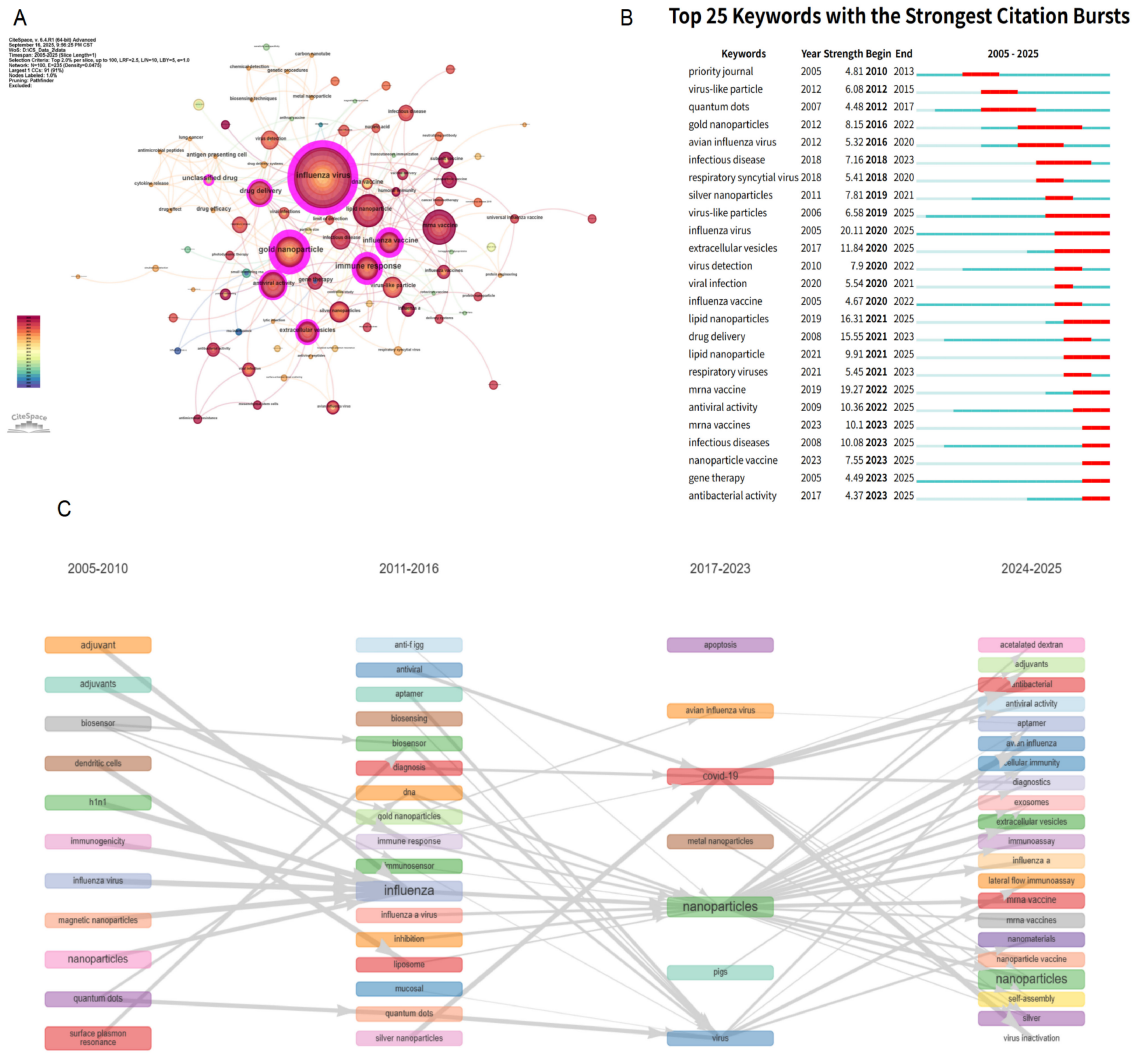
Keywords co-occurrence analysis of nanoparticles in influenza. **(A)** Keywords co-occurrence network. Node size indicates the citation frequency of keywords, and colors represent different clusters. **(B)** Top 25 keywords with the strongest citation bursts. The red line segment representing the duration of high-frequency occurrences and the “strength” indicating the frequency of keywords. **(C)** Dynamic visualization of keyword timeline.

To explore the research focus in the field of NPs and influenza, the top 10 high-frequency keywords were compiled into a table. Since “influenza virus” was one of the search terms, no keyword analysis was performed for it. In recent years (2022–2025), the keyword “lipid nanoparticles” was with 72 occurrence counts and 16.51 surge intensity, “mRNA vaccines” was with 67 occurrence counts and 19.27 surge intensity, “drug delivery” was with 111 occurrence counts and 15.55 surge intensity, and “nanoparticle vaccines” was with 7.45 surge intensity. These keywords indicated that hot topics centered on delivery mechanisms and vaccine development, overlapping significantly with the research priorities reflected in the high-frequency keywords. This suggested that drug delivery technologies and novel vaccine platforms had become the core issues under close scrutiny in this field (**Table 4**; **Figures 8A-C**).

**Table 4:** The top 10 most keywords on nanoparticles and influenza research.

Rank	Keyword	Occurrences
1	influenza virus	310
2	drug delivery	111
3	influenza vaccine	108
4	immune response	75
5	gold nanoparticle	84
6	lipid nanoparticle	72
7	mRNA vaccine	67
8	antiviral activity	51
9	silver nanoparticle	48
10	extracellular vesicles	44

The keyword “drug delivery” had the highest occurrence counts of 111, followed by “gold nanoparticle” with 84 counts, “lipid nanoparticle” with 72 counts, “silver nanoparticle” with 48 counts, and “extracellular vesicles” with 44 counts. These keywords indicated that NPs-based drug delivery is a research hotspot. Advancements in nanotechnology have enhanced the efficacy of existing antiviral drugs for influenza. NPs served as effective carriers for antiviral agents, enhancing ease of administration (22, 23). **Table 5** presents several existing anti-influenza drugs delivered by NPs. The core principle behind nanoscale antiviral therapies lied in exploiting the small size of NPs to facilitate interactions between NPs and viral particles, thus enabling feasible treatment strategies. Additionally, plant-derived NPs and NPs associated with herbal small molecules have gained significant attention. As key components in the preparation of nanodrugs, various nanomaterials have been used to construct NPs incorporating herbal small-molecule compounds, including metal-based NPs such as zinc oxide NPs (ZnO NPs) and AgNPs. β-thujaplicin-modified selenium NPs (SeNPs) exhibit exceptional antiviral activity and serve as effective carriers for anti-influenza drugs (25). **Table 6** lists delivery systems based on traditional Chinese medicine or its components for combating influenza.

**Table 5:** Nanoparticle delivery of anti-influenza drugs.

Drug delivery	NPs material/size	Assembly method	Influenza virus strain	*In vitro*/*in vivo* studies	Reference
amantadine	SeNPs/70-200nm	surface decoration	H1N1	MDCK cells/-	(24)
β-thujaplicin					(25)
oseltamivir					(26)
ribavirin					(27)
oseltamivir	AgNPs/2-3nm	surface decoration	H1N1	MDCK cells/-	(20)
oseltamivir	PDA-NPs/12nm	π-π stacking and hydrogen bonding interactions	H1N1	MDCK cells/female BALB/c mice	(28)

**Table 6:** Research on delivery systems based on traditional Chinese herbal nanoparticles or nanoparticles associated with herbal small - molecule compounds for anti - influenza.

Traditional Chinese medicine or ingredients	NPs Material/Size	Influenza Virus Strain	Research Type	Reference
*Allium cepa*	Fe_3_O_4_ NPs/140nm	H3N2	*In Vitro*: MDCK cells	(29)
*Citrus medica* var. *sarcodactylis Swingle and Limonia acidissima L.*	ZnO NPs/919.9 ± 276.8nm 883.2 ± 68.42nm 2511 ± 70.92nm	H5N1	*In Vitro*: MDCK cells	(30)
*M. bontioides*	Ag-CN NPs/726.93 ± 140.50 nm	H3N2	*In Vitro*: MDCK cells	(31)
*Ellagic acid*	ZnO NPs/202.3 ± 22.6nm	H1N1	*In Vitro*: Vero cells	(32)
*Salvia hispanica*	ZnO NPs/-	H1N1	*In Vitro*: MDCK cells	(33)
*Epigallocatechin gallate*	AgNPs/-	H5N1	*In Vitro*: Vero cells	(34)
*Saccharum officinarum L.*	ZnO NPs/5-30nm	Influenza virus	Computer simulation	(35)
*Cinnamomum cassia*	AgNPs/25-55nm	H7N3	*In Vitro*: Vero cells	(36)
*Houttuynia cordata*	Exosome-Like NPs/169.5nm,166.2nm	H1N1	Computer simulation	(37)
*Raphanus sativus*	ZnO NPs/-	H1N1	*In Vitro*: MDCK cells	(38)
*Panax ginseng*	AgNPs/456nm	H1N1	*In Vitro*: MDCK cells	(39)
*Artemisia annua L.*	Nanovesicles/130nm	H1N1	*In Vitro*: MDCK cells	(40)

The 108 occurrence counts of “influenza vaccine” and 67 occurrence counts of “mRNA vaccine” indicated that NPs in the development of influenza was also significant. The structural design and functional integration of NPs could enhance vaccine immunogenicity and provide broad-spectrum protective effects, representing a core advantage of NPs in influenza vaccine development (41–44). The different types of NPs, each with distinct physicochemical and biological properties, played complementary roles in developing influenza vaccines outlines (**Table 7**). Multivalent epitope NPs, designed to incorporate conserved structural domains of HA, M2e, and NA, provided long-lasting protection against influenza A (H1N1, H3N2) and B viruses in a mouse model (80). NPs could also integrate immune adjuvant functions, such as in the biomimetic NPs, which induces CD8^+^ T cell and mucosal IgA responses through activation of the STING signaling pathway in alveolar epithelial cells, enhancing cross-protection against heterotypic influenza viruses (H5N1, H7N9) (81).

**Table 7:** Different nanoparticles used in influenza vaccine development.

Nanoparticles for vaccines		Antigen target	Route of administration	Challenge Strains	Reference
Virus-like particles	Papaya mosaic virus NPs	NP M2e	i.n. s.c. i.m.	H1N1	(45–48)
	Malva mosaic virus NPs	M2e	s.c.	H3N8	(49)
	Tobacco mosaic virus NPs	HA	s.c. i.m.	H1N1 H3N1 H3N2	(50, 51)
	Bacteriophage NPs	M2e	i.n. i.m.	H1N1 H3N2	(52, 53)
	Mosaic NPs	HA	i.m.	H1N1	(54, 55)
	norovirus protruding particle	NP M2e	i.n. s.c.	H3N2 H1N1	(56, 57)
Biopolymer Particles	Chitosan NPs	HA KAg M2e	i.n.	H1N1 H1N2 H3N2 H9N2	(58–60)
	Self-Assembling Protein NPs	HA M2e	s.c. i.m.	H1N1 H3N2 H9N2	(61–63)
	Polyanhydride NPs	HA KAg	i.n. i.m.	H1N2 H3N8	(64, 65)
Lipid Particles	LNPs	HA M2e NA	s.c. i.m.	H1N1 H3N2 H5N1 IBV	(66–68)
	liposome	M2e HA	i.n. SL	H1N1 H1N2 H3N2	(69–71)
Synthetic Polymers and Inorganic Particles	Poly (lactic-co-glycolic acid) (PLGA) NPs	HA KAg NP M2e	s.c. i.m.	H1N1 H1N2	(72–74)
	Gold NPs	M2e	i.n. i.m.	H1N1 H3N2 H5N1	(75, 76)
	Silver NPs	KLH	i.p.	H3N2	(77)
	Calcium phosphate NPs	HA	i.m.	H1N1 H5N1	(78, 79)

## 4 Discussion

### 4.1 General information

This study collected 3,478 original articles and reviews from the WoSCC, PubMed, and Scopus over the past two decades using predefined search keywords. Using bibliometric software, the research analyzed progress and emerging trends in the application of NPs from various sources for influenza. The field of NPs in influenza research has garnered significant attention and developed rapidly, reflected in the substantial number of related studies and the steady annual increase in publications. Notably, key growth points corresponded closely with global public health events, such as the outbreaks of 2009 and 2020. These public health crises have compelled researchers to turn their attention to social issues that urgently require resolution. NPs technology requires to undergone a transformation from “laboratory innovation” to “social necessity”. This suggested that in the post-pandemic era, NPs technology could be increasingly integrated into public health systems, but its sustainable development will depend on interdisciplinary collaboration and standardized frameworks.

The United States led in the number of 962 publications, with 6 of the top 11 global research institutions based there. In comparison, China ranked second in terms of publication volume of 771 but still had significant room for further exploration. Given the rapid technological advancements, collaborative exchange may be a more effective approach to overcoming scientific challenges. Among the top 10 journals by publication volume, most boasted high impact factors, with Vaccine, published by MDPI, ranking first in terms of the highest number of publications. Masaru Kanekiyo was the most prolific and highly cited author in this field. Bibliometric analysis can help researchers select suitable journals, identify potential collaborators, and understand research hotspots and frontiers, promoting field development.

### 4.2 Research hotspots

The core focus of NPs and influenza has consistently been on vaccine development and the optimization of delivery mechanisms. Further analysis showed that the introduction and application of NPs from various sources have played a significant role in advancing the field. The primary value of NPs in this context lies in three key areas: (1) Antiviral Therapy: exploring the use of NPs carriers for antiviral drug delivery from diverse sources; (2) Vaccine Design and Delivery: optimizing antigen presentation, enhancing immunogenicity, achieving targeted delivery, and enabling controlled release; (3) Influenza Virus Detection: developing highly sensitive and specific nanoscale sensors and diagnostic platforms. Research in these three areas not only deepened the theoretical understanding of NPs applications in influenza prevention and control but also enhanced their practical application potential.

### 4.3 Antiviral therapy of nanoparticles

Based on their source, NPs can be classified as either organic or inorganic and loaded with natural bioactive compounds or medicinal plants to reduce side effects (82, 83). Metal NPs serve as inorganic and effective carriers for antiviral drugs, including amantadine, β-cyperone, oseltamivir, and ribavirin, effectively inhibiting the replication of influenza viruses (24–27). Hussein Hadi Mossa Mishbak improved the antiviral activity of Fe_3_O_4_ NPs by enhancing targeted delivery through biosynthetic integration of quercetin, extracted from onions (29).

Plant-derived vesicle-like NPs, produced naturally by plants as organic carriers, have gained significant attention due to their biocompatibility, safety, cost-effectiveness, low immunogenicity, and easy availability in recent years (84, 85). Lusha Ye prepared nanovesicles from *Artemisia annua* to enhance the stability of gamma-aminobutyric acid encapsulated within the vesicles, allowing efficient delivery to lung and alleviation acute lung injury induced by the influenza virus *in vivo* administration (40). Extensive research has indicated that traditional Chinese herbal medicine exhibits significant efficacy in antiviral treatment. The root of *Isatis indigotica Fort* and the flower of *Lonicera japonica Thunb* were among the most renowned medicinal herbs used to treat IAV infections (86, 87). The combination of Chinese herbal medicines with NPs may be a key area of future research and development.

### 4.4 Vaccine design and delivery

NPs have made significant contributions to influenza vaccine development (88). Vaccination remains an efficient and cost-effective strategy for controlling influenza epidemics and maintaining public health. Since its introduction in the 1940s, seasonal influenza vaccines have saved countless lives and reduced the spread of pandemics (89). However, influenza viruses continuously evolve through genetic mutations to evade natural immunity, requiring regular updates to vaccines. Current influenza vaccines primarily use inactivated or attenuated live viruses, both of which have significant limitations (90, 91).

Among various types of nanoplatforms, virus-like particles (VLPs) are self-assembling, non-replicating structures composed of viral capsid proteins. Vaccine platforms based on plant viral capsid proteins have a long history and demonstrate significant efficacy. Examples include Papaya mosaic virus (45–47), Malva mosaic virus (49) and Tobacco mosaic virus (50, 51), which primarily function by conjugating their capsid proteins with epitopes or immunodomain regions. The prominent (P) domain of norovirus exhibited stability and high immunogenicity, making it an excellent vaccine antigen (92). Ming Xia constructed a P particle-M2e chimeric vaccine, and mice vaccinated with this formulation exhibited strong immune responses to M2e and survived lethal influenza virus challenges (57). By simulating the structure of natural viruses, VLPs could enhance the antigenicity of attenuated viral vaccines while retaining the advantages of recombinant subunit vaccines (93).

Biodegradable and biocompatible polymer NPs represent a significant research focus in the 21st century, particularly as efficient carrier systems. Ferritin, a naturally occurring protein, could spontaneously form protein cage structures to delivery antigen in a virus-like manner, offering enhanced controllability and stronger immunogenicity (94). Nicole Darricarrère immobilized a head-removed HA stable stem immunogen on ferritin NPs, which have induced broad-spectrum neutralizing antibody responses against diverse H1 and H3 viruses (95). Beyond proteins, Chitosan, a naturally occurring polysaccharide that was non-toxic, bioadhesive, biodegradable, and biocompatible, has been widely utilized as a carrier for peptides, proteins, and DNA-based vaccines. Seyed Farid Sadati used chitosan NPs as a degradable delivery system for low-dose antigen molecules, potentially reducing vaccine production costs significantly (96).

Amphiphilic lipid-based NPs could function as artificial transport carriers for influenza antigens, facilitating antigen delivery into cells (97). Xinyu Yue utilized clinically validated and safe LNPs to deliver circRNA-encoded antigens. The results showed that this safe vaccine effectively protected mice against multiple influenza strains (67). The primary advantage of polymer NPs formulations lies in the controlled release of encapsulated antigens and/or adjuvants (98). Amir Tukhvatulin encapsulated influenza HA antigens in Poly (lactic-co-glycolic acid) (PLGA) NPs and combined them with a TLR4 agonist, enhancing both humoral and cellular immunity in mice (74). Metal-based NPs, which can chemically conjugate antigens and/or adjuvants to their surfaces, offer the benefit of enhancing antibody titers through surface antigen presentation (98). AuNPs, known for their small size, excellent biocompatibility, and stability, have become widely applied in the development of universal influenza vaccines (75, 76).

Beyond conventional vaccine design approaches, encapsulating herbal extracts within NPs as vaccine adjuvants could be a highly promising research direction. Pengfei Gu utilized PLGA NPs encapsulating *Angelica sinensis* polysaccharides, demonstrating greater potential to stimulate both humoral and cellular immune responses compared to aluminum-based and oil-based adjuvants (99). Fernando Silveira discovered that *Quillaja brasiliensis* saponin-based nanoadjuvants effectively enhance the humoral immune response to influenza vaccines in aged mice. Although research on herbal-derived nanoadjuvants remains limited, they hold significant potential to become a major focus of future studies (100, 101). Overall, future research should rationally select or combine different NPs platforms based on vaccine design objectives to overcome the limitations of traditional influenza vaccines.

### 4.5 Influenza virus detection

Influenza diagnostic technologies primarily included polymerase chain reaction (PCR), enzyme-linked immunosorbent assay, reverse transcription PCR, and immunofluorescence assays (102). To broaden the range of detection methods and achieve rapid, fully automated techniques with high sample throughput potential, various nanomaterials have been employed in diverse combinations to detect influenza virus (103). Noble metal NPs, with optical, electrical, chemical, and biological properties that depend on their size and shape, have gained increasing attention in recent years (104, 105). AuNPs are extensively studied and applied due to their high stability (106). In their spherical form, AuNPs exhibit excellent selectivity and sensitivity, making them ideal for the fabrication of colorimetric sensors (107). Jinhua Wei used AuNPs functionalized with glycan clusters to assess the interaction between HA and host glycan receptors via colorimetric assays, facilitating the development of a simple and sensitive system for identifying the receptor-binding properties of influenza viruses (108). AgNPs were also widely used in photonics and sensor applications, with modified AgNPs showing particular effectiveness in DNA detection. Their high electrical conductivity and stability lead to superior detection performance (109). Yan Xu’s team developed a homogeneous nucleic acid assay using single-particle inductively coupled plasma mass spectrometry with AuNP and AgNP probes, enabling simultaneous sensitive detection of both novel coronavirus and influenza viruses (110). Additionally, magnetic NPs, silica NPs, and europium NPs have demonstrated value as nanomaterials for influenza detection. The key advantage of nanomaterial detection technology lies in its multifunctionality and flexibility, owing to the vast potential for NPs surface modification.

### 4.6 Limitations of research methods

This study employed bibliometric methods to systematically analyze the progress and key issues in the field of nanomaterials for influenza research, providing an overview of the current state of research. However, this study has certain limitations. First, due to limitations in the file format exported from PubMed, only the WoSCC and Scopus databases were analyzed for reference analysis. Second, the study sample only includes research papers and review articles published in English, which may introduce language bias. Furthermore, given the dynamic nature of the WoSCC database, some recently added literature may not be fully covered in this study.

Additionally, CiteSpace, VOSviewer, and Bibliometrix bibliometric software are commonly used in bibliometrics and scientific knowledge mapping. CiteSpace employs conventional data similarity algorithms, utilizing set theory concepts to visually represent the similarity between two datasets (111). VOSviewer employs an association strength algorithm that normalizes co-occurrence data using probabilistic principles (112). Bibliometrix, an open-source tool programmed in R, features robust and efficient statistical algorithms, high-quality numerical algorithms, and rapid upgrade capabilities (113). All three software tools can enable scientific computation and descriptive analysis of bibliometric data, support network analysis, and visualize networks intuitively. Their core positioning is consistent, and the core analytical functions overlap partially.

### 4.7 Future trends and challenges

Recombinant biotechnology combined with NPs can enhance immunogenicity and direct immune responses to target conserved antigenic sites on viruses, enabling the development of influenza vaccines with broad protective efficacy. This represents an ideal goal for NPs-based vaccine development. Whether utilizing VLPs, biopolymers, liposomes, or synthetic particles, various research approaches in this field have already led to promising strategies. The field is expected to continue expanding as vaccines improve over time.

The application of traditional Chinese herbal medicine in combating influenza is on the rise, though studies exploring its combination with NPs remain relatively limited. Currently, no universal standard protocol exists for the characterization or quality control of plant-derived nanovesicles (PDNVs), as their morphology, size, and active compound concentration can vary based on plant species, tissue origin, or environmental factors. This variability makes it technically challenging to obtain high-purity, high-yield PDNVs (114, 115). It is recommended to develop general principles tailored to the plant-specific characteristics to create a framework for diversifying plant sources. PDNVs are prone to aggregation and degradation over time, reducing their efficacy and shelf life (115). Thus, further research on their long-term stability is needed. Meanwhile, phytochemicals hold promise as synergistic models for future biomedical applications, owing to their potential in creating novel drugs and synthetic NPs.

In viral detection, nanotechnology has enabled high sensitivity and specificity in detecting specific viruses. The use of nanocarriers that combine diagnostic and therapeutic functions has further enhanced the sensitivity, stability, and response time of detection methods. However, few tests have been conducted using actual samples from infected patients. The transition from research to routine clinical applications remains a significant challenge. The main limitation lies in the interference from substances found in real-world samples such as blood, urine, or saliva, which necessitates extensive modifications to the surface of NPs.

## 5 Conclusion

This study has provided an indepth analysis of research directions and future prospects of NPs applications in influenza research. The analysis of bibliometric can help researchers select appropriate journals for publication, identify potential collaborators, and understand research hotspots and frontier areas, thereby advancing the field. Overall, research on NPs in influenza is undergoing rapid development. The findings indicate that the primary focus of the field is the application of NPs in delivery optimization and vaccine development, while also exploring the use of diverse NP sources and their combination with natural herbs or compounds. 

## Data Availability

Publicly available datasets were analyzed in this study. This data can be found here: https://pubmed.ncbi.nlm.nih.gov/; https://webofscience.com/wos/woscc/basic-search; https://www.scopus.com.
